# Effect of a combined program of running exercise and environmental enrichment on memory function and neurogenesis markers in amyloid-beta-induced Alzheimer-like model 

**DOI:** 10.22038/IJBMS.2023.70269.15277

**Published:** 2023

**Authors:** Mahsa Saheb, Mohammad Amin Khodadadegan, Sajad Sahab Negah, Ehsan Saburi, Vahid Hajali

**Affiliations:** 1 Department of Neuroscience, Faculty of Medicine, Mashhad University of Medical Sciences, Mashhad, Iran; 2 Student Research Committee, Faculty of Medicine, Mashhad University of Medical Sciences Mashhad, Iran; 3 Neuroscience Research Center, Mashhad University of Medical Sciences, Mashhad, Iran; 4 Medical Genetics and Molecular Medicine Department, Faculty of Medicine, Mashhad University of Medical Sciences, Mashhad, Iran

**Keywords:** Alzheimer’s, Combination effect, Environmental enrichment, Exercise, Neurogenesis, Spatial memory

## Abstract

**Objective(s):**

It is urgent to develop non-pharmacological interventions or multifactor combination approaches to combat Alzheimer’s disease (AD). The effect of exercise (EX) combined with environmental enrichment (EE) on behavioral phenotypes and neurogenesis markers in an Alzheimer-like rat model was investigated.

**Materials and Methods:**

The groups consisted of AD, sham-operated, AD+EX, AD+EE, and AD+EX+EE. AD was produced by injection of amyloid-beta (1-42, 6 µg) intrahippocampally, and a daily treadmill for 3 consecutive weeks was used for EX animals. EE was a large cage (50× 50× 50 cm) containing differently shaped objects. Spatial learning and memory were evaluated in the Morris water maze (MWM), and a shuttle box was used to evaluate inhibitory avoidance memory. RT-PCR was performed to assess the expression of early neurogenesis markers, DCX, and Sox2 within the hippocampus.

**Results:**

Pretreatment with exercise and EE, both individually and in combination, could provide protection from memory impairments in AD rats. Combined treatment led to a significantly more pronounced improvement in memory deficits of AD rats than either paradigm alone. Combination therapy with exercise and EE could also reverse the passive avoidance memory impairment and hippocampal DCX expression of AD rats to the control levels.

**Conclusion:**

These data suggest that exercise in combination with cognitive engagement can provide a non-pharmacological and multidomain policy that may prevent or delay AD symptoms.

## Introduction

Alzheimer’s disease (AD) is a progressive and chronic brain disorder that leads to the gradual loss of cognitive, reasoning, abstraction, language, and behavioral skills, ultimately resulting in dependence on caregivers for daily living activities. The increasing population, longevity, and financial prosperity have raised concerns about widespread dementia among the aging population in the coming decades. At present, there are 26 million AD patients worldwide, and the number is estimated to reach around 106 million by 2050, raising serious ethical, economic, clinical, and social concerns (1, 2). AD is a multifactorial syndrome that causes a gradual decline in brain functioning due to a complex variety of neurochemical alterations, including changes in synaptic density and function, neurogenesis, and the formation of beta-amyloid (Ab) plaques, neurofibrillary tangles (NFTs), neuroinflammation, oxidative stress, and so on (3). The disease has been recognized for more than 100 years, but a definitive treatment strategy has not been established yet due to its uncertain etiology and complex pathogenesis. Currently, acetylcholinesterase inhibitors (AChEIs) such as donepezil, rivastigmine, galantamine, and NMDA receptor antagonist memantine offer only temporary symptomatic relief. Therefore, there is an urgent need to explore the effectiveness of non-pharmacological interventions such as cognitive or physical practices or combined approaches to combat this disabling disease.

A healthy lifestyle, including a balanced diet, regular exercise, and engagement in activities that involve socializing and stimulating the brain, has been suggested to have beneficial effects on cognition and reduce the overall risk of developing AD (4, 5). The positive effects of exercise on brain health and cognitive functioning have been well-documented in both human and animal studies (6, 7). Physical exercise has been demonstrated to improve brain function and memory through multiple mechanisms, including improving cerebrovascular status, angiogenesis, neurogenesis, synaptic plasticity, and growth factor secretion (7, 8). For decades, researchers have investigated physical exercise as a behavioral intervention to mitigate neurological impairments in experimental studies. Regular physical exercise has been shown to delay, ameliorate, or even prevent cognitive decline in elderly people (9, 10), aged rats (11), or AD transgenic mice (12).

Numerous epidemiological studies have highlighted poor cognitive stimulation as a risk factor for cognitive decline development. Higher educational and occupational attainment has been consistently associated with a lower risk of developing dementia in general and AD in particular (13, 14). In experimental studies, active lifestyle behavior can be modeled by housing multiple rodents in a large chamber where a variety of complex and challenging objects (tunnels, toys, shelter, ladder, nesting materials) and a voluntary running wheel are present to provide a stimulating environment (enriched environment; EE) for sensory, cognitive, motor, and social development compared to standard housing (15). Several studies have reported the positive effects of EE on both cognitive and biochemical features of neurodegenerative pathology in experimental models (16, 17). EE increases the expression of neurotrophic factors and other molecules associated with synaptic plasticity, neurogenesis, axonal transport, and dendritic branching, exerting strong neuroprotective effects (16, 18, 19). Although conflicting data is showing that the effect of EE on AD pathology, neurogenesis, or cognitive performance is limited and variable (17, 20-23), EE has recently gained attention as a potential non-pharmacological strategy that might affect the onset and progression of neurodegenerative diseases, including AD.

In recent times, there has been a growing interest in combining various interventions to improve cognitive performance, which has shown great promise as a therapeutic approach for treating various health conditions. Particularly, the combination of physical and cognitive training has attracted increasing interest. The human data analysis revealed that combined physical and cognitive training has an advantage over training alone in terms of cognition and brain structure and function (24, 25)post-intervention standardized mean difference (SMD. The same advantage of this combination has also been reported in terms of various aspects of neural plasticity events and cognitive tasks in animal models (26, 27)the efficacy of long term exposure of VSL rats to combination paradigm of environmental enrichment (EE. In our previous study, we found a superior function in memory along with greater hippocampal expression of the molecular marker of memory in a combined program of treadmill running and EE than either treatment individually in healthy rats (28). However, to date, the data on the combination of EE and physical activity for cognitive deficits in neurodegenerative status is scarce. 

With these promising data concerning the additive effect of simultaneous exposure to EE and physical exercise on cognition in non-neurodegenerative status, we asked whether simultaneous exposure to two paradigms in amyloid beta-induced AD rats may also have a greater impact on learning and memory function than either treatment alone. In addition, we aimed to investigate if neuronal development plays a role in our findings. This was achieved by assessing the levels of DCX and Sox2, which are markers of early hippocampal neurogenesis in the hippocampus of rats.

## Materials and Methods 

***Animals***

**Figure 1:**
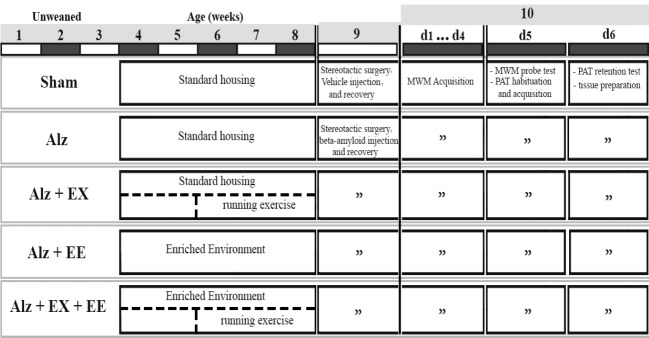
Experimental design of the study

The experiments and animal handling procedures were conducted in accordance with the guidelines set by the Laboratory Animal Ethics Committee of Mashhad University of Medical Sciences (IR.MUMS.MEDICAL.REC.1398.421). Every effort was made to minimize the number of rats used and any potential suffering. The timeline of the experiments is presented in Figure 1. Male rat pups were acquired from the colony maintained by the Animal Facility at Faculty of Medicine of Mashhad University of Medical Sciences, Iran.

***Experimental design***

At one month of age, the animals were randomly assigned to one of the following five experimental groups, each consisting of eight individuals (n=8): i) Alzheimer: Rats received bilateral intrahippocampal injections of amyloid-beta (6 µg/6µl) and were housed in standard animal cages measuring 50*30*25 cm. ii) Sham-operated: Rats received the same volume of vehicle solution via the same route and were kept in standard cages. iii) Alzheimer+Enriched Environment (Alz+EE): Rats were housed in larger cages measuring 50*50*50 cm for a duration of 5 weeks. These cages were furnished with nesting materials, tunnels, ladders, shelters, houses, and toys, which were regularly modified and rearranged to promote a sense of novelty. Subsequently, the rats received intrahippocampal injections of Aβ 1-42. iv) Alzheimer+Exercise (Alz+EX): Rats in standard cages underwent treadmill running sessions for a period of 3 weeks, five days a week, and were then subjected to intrahippocampal injections of Aβ 1-42. v) Alzheimer+EX+EE: Rats were exposed to 5 weeks of an enriched environment combined with 3 weeks of treadmill exercise, followed by intrahippocampal injections of Aβ 1-42 (as shown in Figure 1). Behavioral tests were initiated when the animals reached 2 months of age. After the completion of behavioral tests, the rats were euthanized for hippocampal dissection and subsequent analysis (refer to Figure 1). The animals were housed in groups of four and eight in standard and enriched cages, respectively. They had unrestricted access to food and water under all conditions and were kept in a climate-controlled room maintained at 23 ^°^C ± 1 ^°^C, following a 12-hour light-dark cycle (lights on from 06:00 to 18:00 hours). All cages were cleaned once a week. 

***Intrahippocampal microinjection of Aβ 1-42***

**Figure 2:**
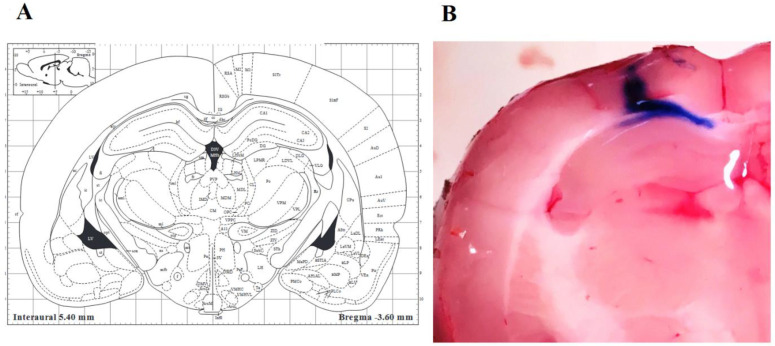
(A) Schematic representation of the rat brain coordination from Paxinos atlas. (B) The ink was injected stereotaxically into the CA1 area in the pilot experiment to assure the correct coordination

The animals were administered anesthesia with ketamine-xylazine (100-10 mg/kg, IP; Vibac Laboratories, Carros, France) and secured in a stereotaxic frame. Burr holes were drilled bilaterally in the skull, targeting the CA1 region of the hippocampi, using the following coordinates: 3.6 mm posterior to bregma, 2.4 mm lateral to the sagittal suture, and 3.6 mm ventral to the surface of the skull (refer to Figure 2). Aβ 1-42 (6 µg in 6μ PBS) or vehicle was bilaterally injected (3 µl per side) through a 27-gauge injection needle, which was connected to a Hamilton syringe (10 µl) via a polyethylene tube. The injection was manually administered over a period of 10 min, and the injection needle was left in place for an additional 60 sec to optimize diffusion away from the needle tip and minimize dorsal diffusion.

***Treadmill exercise***

In the exercise groups, the rats were subjected to treadmill running once a day for 5 consecutive days per week over a period of 3 weeks. The specific details of the treadmill regimen were as follows. During the first two weeks, the rats ran on the treadmill at a speed of 3 m/min for the initial 5 min, followed by an increase to 6 m/min for the subsequent 5 min, and finally reaching a speed of 10 m/min for the final 20 min. In the third week, the running speed was adjusted. The rats ran at a speed of 6 m/min for the first 15 min, then increased to 10 m/min for the next 15 min, and finally reached a speed of 15 m/min for the last 15 min. Each session in the third week included a 5-minute break for the animals. Throughout all the treadmill sessions, the inclination of the treadmill remained at 0%. Additionally, the rats received a mild shock of 0.25 mA whenever they stopped running, to encourage continuous movement. All treadmill sessions were conducted during the light cycle between 9:00 and 14:00. This modified protocol was based on previous studies by Saadati *et al*. (29) and Kim *et al*. (30).

***Spatial learning and memory in morris water maze (MWM)***

In the Morris Water Maze (MWM) experiment, a circular tank was used, measuring 160 cm in diameter and 80 cm in height. The tank was filled with 22-24 ^°^C water, with a depth of 50 cm. To divide the pool into quadrants, it was conceptually separated into four equal parts. Each quadrant was assigned a number: 1, 2, 3, and 4. In the center of quadrant 3, a round platform measuring 10 cm in diameter was positioned 2 cm below the water’s surface, hidden from the view of the rats. The trials took place in a dimly lit room, where various fixed images (such as squares, circles, or triangles) were attached to the walls surrounding the maze. An automated video tracking system (Borj Sanat Azma) recorded the rats’ performance. The MWM experiments spanned five consecutive days. During the first four days of training, each rat completed four trials daily, with a 5 min break between each trial. In each trial, the rat was released into the water from one of the four quadrants, facing the wall of the pool. Throughout the training trials, the location of the platform remained constant, allowing the rats to search for the hidden escape platform within a time limit of 60 sec. Once the rat found the platform, it stayed on it for 15 sec before being removed from the water, dried with a towel, and returned to its home cage until the next trial. If a rat failed to find the platform within the allotted time, the experimenter manually guided it to the platform, following the same procedure as the others. To assess spatial learning, data on the time taken and distance covered to reach the hidden platform were collected and analyzed as criteria. On the fifth day, a single probe trial was conducted, 24 hr after the final training trial, to evaluate spatial memory. In this trial, the rat was released into the pool without the presence of an escape platform and allowed to swim freely for 60 sec. The time spent, distance traveled, and the number of crossings over the target quadrant were examined as parameters for spatial memory. All behavioral procedures for the various groups were conducted during the lights-on period, ensuring consistent conditions throughout the experiments.

***Passive avoidance task***

The passive avoidance task was conducted using a shuttle box apparatus consisting of two chambers of equal dimensions (25×25×20 cm) connected by a guillotine door. One chamber was brightly illuminated, while the other was dark. The floor of both chambers was made of stainless-steel grids, capable of delivering electric shocks (50 Hz, 3 sec, and 1 mA intensity) through a standard stimulator. The passive avoidance task assesses long and short-term memories in an associative manner, utilizing fear motivation. Taking advantage of rodents’ natural preference for the dark compartment, this task evaluates inhibitory avoidance memory. The animal learns to associate the dark chamber with a prior aversive stimulus. The task was conducted over two consecutive days. On the first day (habituation and acquisition phases), the animal was initially placed in the light chamber with the guillotine door opened after 5 sec, allowing free exploration of both chambers for 5 min. Thirty min later, the animal was placed in the bright chamber, and after 5 sec, the guillotine door was opened, enabling the animal to freely move to the dark chamber. Animals with an initial latency to enter the dark chamber exceeding 100 sec were excluded from the study. Once the animal entered the dark chamber with all four paws, the door was closed, and the animal remained there for 10 sec before being returned to its home cage. After an interval of 1 hr, the animal was placed back into the light chamber, and the door was opened after 5 sec. This time, upon entering the dark chamber, the door was immediately closed, and a weak electric shock (1 mA, 50 Hz, 3 sec) was delivered to the animal’s foot through the steel grids on the floor. After 10 sec, the animal was returned to its home cage and kept there for 120 sec. The same procedure was repeated for subsequent sessions. Successful acquisition of inhibitory avoidance memory was defined as the animal staying on the light side without moving to the dark side for at least 120 sec. However, if an animal moved to the dark chamber within 120 sec, the guillotine door was closed, and another shock was administered as in the first trial. On the second day (retention phase), the latency to enter the dark chamber without the presence of an electric foot shock was measured, following the same procedure as the acquisition trial. The step-through latency (STL), the number of entries into the dark compartment, and the total time spent in the dark compartment (TDC) were recorded as retention parameters, with a maximum STL cutoff of 300 sec.

***Tissue dissection and real-time PCR***

Following the completion of behavioral experiments (Figure 1), the animals were decapitated, and both whole hippocampi were swiftly extracted and frozen in liquid nitrogen. They were then stored at -80 degrees Celsius until further homogenization. To evaluate the mRNA expression of early neurogenesis markers, DCX and Sox2, in the hippocampus, quantitative real-time PCR (qRT-PCR) was conducted. Total RNA was extracted using the RNeasy Mini kit (Parstous, Iran), and the concentration of the RNA was measured at 260 nm absorbance using a nanodrop spectrophotometer (Thermo Fisher Scientific, Germany). First-strand cDNA was synthesized from 1000 ng of RNA using a cDNA Synthesis Kit (Yekta-Tajhiz; Cat: Yt4500), following the manufacturer’s instructions. qRT-PCR amplification was performed using the CFX 96 Real-Time System (Roche Applied Science, USA) and Syber Green dye (Amplicon, Denmark). All qRT-PCR reactions were performed in duplicate. The relative expression levels of the target genes were calculated using the 2-ΔΔCq (Livak) method, with normalization to the internal control (β-actin). 

***Statistical analysis***

The time and distance required to locate the hidden platform during the MWM training days in the separate groups were subjected to repeated-measure analysis. To compare these variables between the groups, a two-way ANOVA with repeated measures (group and block as factors) was conducted. For the analysis of data obtained from the probe trials, swimming speed, passive avoidance variables, and gene expression, a one-way ANOVA followed by Tukey’s *post-hoc* multiple comparison tests was employed. Non-parametric data were analyzed using the Kruskal-Wallis test, followed by the Bonferroni adjustment. The values are presented as means±SEM and statistical significance was set at *P*<0.05. 

## Results

***Spatial learning and memory in MWM***

**Figure 3:**
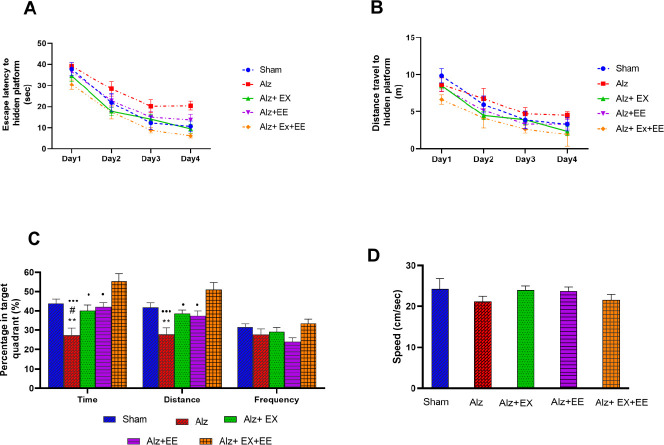
A, B: Spatial learning in the Morris water maze (MWM) test in sham, Alz (Alzheimer), Alz+EX (exercise), Alz+EE (enriched environment), and Alz+EX+EE rats

During the acquisition trial days, it was observed through repeated measures analysis that all singular groups were able to learn the location of the hidden platform, which was evident by the decrease in escape latency and distance traveled over the course of four subsequent training days (*P*=0.000). The analysis of repeated measures ANOVA did not reveal any significant differences among the groups in terms of escape latency or distance traveled (*P*=0.892 for time and *P*=0.781 for distance, as shown in Figures 3A and B). The spatial memory parameters in the single probe trials are depicted in Figure 3C. One-way ANOVA showed significant differences in the percentage of time (F(4,35)=10.45, *P*=0.000) and distance (F(4,35)=9.16, *P*=0.000) spent in the target quadrant (Q3) among the groups. Tukey’s *post-hoc* test revealed that the Alz group spent significantly less time in the target quadrant as compared to the sham, Alz+EX, Alz+EE, and Alz+EX+EE groups (*P*<0.005, 0.05, 0.05, and 0.001, respectively). Moreover, the decrease in the percentage of distance in the Alz group was found to be significant only when compared to the sham and Alz+EX+EE groups (*P*<0.005 and 0.001, respectively). Interestingly, the animals in the Alz+EX+EE group spent more time and distance in the target quadrant as compared to both the Alz+EX and Alz+EE groups (*P*<0.05). Furthermore, there was no significant difference observed in the MWM swimming speed among the five experimental groups (as shown in Figure 3D).

***Passive avoidance memory in the shuttle box***

**Figure 4:**
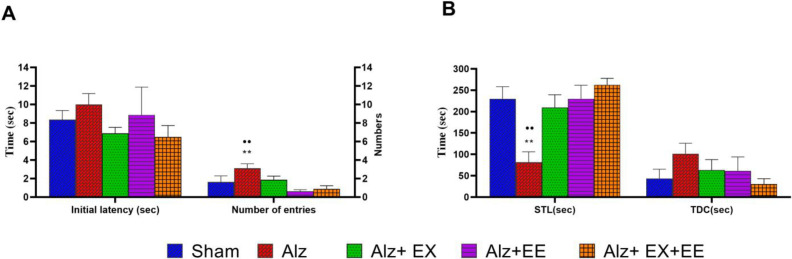
A, B: The passive avoidance memory in the shuttle box apparatus in sham, Alz (Alzheimer), Alz+EX (exercise), Alz+EE (enriched environment), and Alz+EX+EE rats

The results of the passive avoidance memory are shown in Figures 4A and B. The Kruskal-Wallis test revealed a significant difference in STL (*P*=0.011) and the number of entries (*P*=0.010) between the groups. After the Bonferroni adjustment, it was found that STL is significantly decreased in the Alz group compared with the sham group (*P*=0.004). Comparison between the Alz and Alz+EX+EE groups showed that the latter group had significantly longer STL than the former one (*P*=0.002, Figure 4B). The number of entries to the dark part in the Alz group was significantly increased compared to the Alz+EE (*P*=0.001) and Alz+EX+EE (*P*=0.004) groups (Figure 4A). The remained differences did not meet statistical significance. 

***DCX and Sox2 mRNA expression***

**Figure 5:**
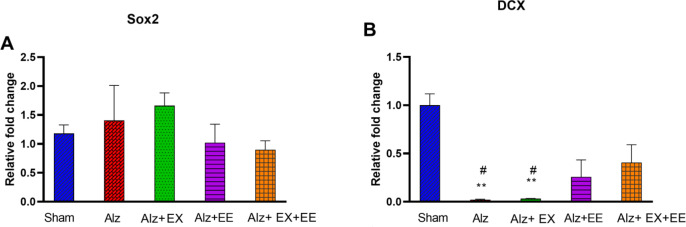
A, B: Hippocampal gene expression of early neurogenesis markers, Sox2 (A) and DCX (B) in sham, Alz (Alzheimer), Alz+EX (exercise), Alz+EE (enriched environment), and Alz+EX+EE rats

Figures 5A and B show the results of Sox2 and DCX mRNA expression in the hippocampus of rats. The mRNA expression of Sox2 was the same in all groups (*P*=0.496, Figure 5A). One-way ANOVA revealed a significant difference in hippocampal DCX between the groups (F_(4,10)_= 12.40, *P*=0.001, Figure 5B). Tukey’s *post-hoc* analysis showed that DCX is severely decreased in Alz and Alz+EX groups compared to the sham group (*P*<0.005). DCX expression was significantly reversed when Alz animals were submitted simultaneously to exercise and EE in the Alz+EX+EE group (*P*<0.05 in comparison with Alz and Alz+EX groups). The hippocampal Sox2 levels were the same in all groups. The remaining differences did not meet statistical significance.

## Discussion

Regarding the positive impact of physical activity and EE, both individually and in combination with other paradigms on cognitive functions and with respect to consistent neuronal mechanisms through which exercise and EE affect cognition; the current study was designed to examine whether a combined program of physical exercise and EE would yield a greater protective effect than either treatment alone against the cognitive deficits of AD rats. Animals that were injected with amyloid-beta exhibited a decline in their spatial memory abilities, as evidenced by reduced time spent and distance traveled in the target quadrant of MWM. Pretreatment with running exercise and EE, both individually and in combination, could provide protection from memory impairments in AD rats. Exercise and EE in combination led to a significantly more pronounced improvement in memory deficits of AD rats than in either paradigm alone. Passive avoidance memory was also disrupted in AD animals as revealed by the decrease in retention time to enter the dark chamber. While pretreatment with both individual and combined exercise and EE increased the retention time toward the control levels, the combined paradigm was only significantly efficient. Concerning neurogenesis marker analysis, amyloid-beta injection led to a significant decrease in the mRNA expression of hippocampal DCX, and combination pretreatment with exercise and EE could significantly restore it. 

AD is a prevalent and devastating neurodegenerative condition that is characterized by the accumulation of amyloid plaques, the formation of neurofibrillary tangles, significant neuronal loss, and a decline in cognitive function (31). Despite the disease representing a global public health and social care crisis, there are still no promising treatment strategies available. Both the familial and sporadic forms of AD express common neuropathological hallmarks, suggesting that different causes, factors, and pathways could be involved in the neuropathological deficits of the disease. Experimental animal models of AD display growing and persistent memory deficits which are analogous to the symptoms of human sporadic AD (32). Our data were in accordance with previous studies indicating that ICV or intrahippocampal microinjection of amyloid-beta causes memory disturbances. However, it should be noted that a single injection of amyloid peptide does not exactly mimic all of the pathological features and neurochemical components of AD. While to date no ideal animal model that reproduces all of the features of AD in humans has been developed, the genetic mouse models and amyloid-beta microinjection are the most relevant existing experimental AD models. Injecting amyloid-beta into the brain of rats triggers oxidative stress, pro-inflammatory responses, and a sequence of neurotoxic events that ultimately result in damage to the neuronal functions responsible for the behavioral symptoms associated with AD. Therefore, the injection of amyloid-beta into the rat brain is considered an acceptable model to induce some neural and behavioral features of AD analogous to those seen in transgenic animals (33). 

Research studies investigating the potential benefits of exercise and EE have typically focused on exploring these therapies after experimentally-induced cognitive and brain function deficits have occurred (i.e., post-treatment) (26, 34). However, some studies have also employed a pretreatment approach with exercise and EE, administering the therapies before the experimental induction of AD-like pathology in animals (34, 35). Voluntary exercise plus cognitive stimulation in the form of EE beginning before disease onset and continuing during the disease course counteract memory decline and anxiety-like behaviors in AD transgenic mice (36, 37). A study by Herring *et al.* (38) compared the preventive and therapeutic effects of EE on beta-amyloid pathology in transgenic mice and concluded that both approaches are capable to reduce the beta-amyloid burden, though with the different plaque and cerebral angiopathy morphology. The prophylactic viewpoint of exercise and cognitive training has also been established in human studies. Among many lifestyle behaviors that could influence the course and pathology of AD, physical exercise is one of the most important habits that may assist in preventing AD or slowing the decline of cognitive components in patients with mild to severe AD (39, 40). There is mounting evidence to suggest that engaging in cognitive activities before the onset of dementia and AD can reduce the risk of developing these conditions by increasing cognitive reserve (4, 41, 42). In our study, we aimed to evaluate the potential preventive and prophylactic effects of combination therapy of exercise and EE on cognitive impairment and related molecular targets.

Both physical exercise and EE have been independently well-documented to improve cognitive scores in experimental healthy subjects as well as in cognitively impaired models (11, 43, 44). Treadmill exercise ameliorated memory impairment and increased hippocampal dendritic length, neurogenesis, and BDNF expressions in the amyloid-beta injected rats, suggesting that exercise may have therapeutic benefits for relieving symptoms of AD (45). Dao *et al.* (46) also showed that regular exercise could prevent deficits in short-term memory and hippocampal long-term potentiation (LTP) in a rat model of AD (i.c.v amyloid-beta infusion). A recent study reviewed the protective capacity and corresponding mechanisms of treadmill exercise in different models of rodent memory deficits, particularly in AD-induced models (47). EE has also attracted much interest as a potential noninvasive approach that might affect the onset and development of neurodegenerative disorders including AD. EE has been shown to reduce memory deficits and neuropathological hallmarks in animal models of Alzheimer-like neurodegeneration (19, 34).

Consistently, these interventions in the present study could separately recover the impaired spatial memory of amyloid-beta-injected animals. While it is widely recognized that treatment with exercise or EE individually can partially alleviate the pathogenic phenotypes of the AD brain, there is limited information available on whether the combination of these two paradigms can work synergistically to improve pathogenic phenotypes in animal models that exhibit human-like AD symptoms. Therefore, the primary objective of this study was to examine whether cotreatment with exercise and EE is more efficient in restoring cognitive functions in AD rats than either paradigm alone. Results showed that while both exercise and EE separately could restore spatial memory performance in AD rats, combination therapy was significantly more effective. It seems that this augmented effect of combination therapy was additive but not synergistic, with each treatment alone producing significant improvement that when combined led to more efficacy. During acquisition, all individual groups were trained successfully; however, the Alzheimer group showed a slight increase in the acquisition blocks that did not meet the significance level. Given this, and considering the same swimming speed among the groups, it could be expected that the observed changes in spatial memory function may not be due to the potential confounding issues such as different sensorimotor processing or motivation of the animals.

Exercise and EE individually in combination with a variety of treatments have been evaluated to achieve a greater advantage for neurobehavioral dysfunctions in AD animal models. The effects of physical training in combination with antioxidants (α-lipoic acid) have been investigated on the pathological phenotype of transgenic AD mice. This study found that compared to the control and single treatment groups, 16 weeks of treadmill running combined with lipoic acid in AD mice could ameliorate spatial cognitive deficits and protect the neuronal injury induced by amyloid-beta deposition (48). Another study also showed that a combination regimen of involuntary treadmill running, voluntary wheel exercise, and acousto-optic stimulation could potentially reserve the reduced hippocampal neurogenesis and impaired behavioral functions in a mouse model of AD (49). Regarding EE, Dong *et al*. (50) examined the effects of combination therapy with EE and memantine (NMDA receptor antagonist approved for the treatment of AD) on cognitive functions and AD-like pathology of senescence-accelerated prone mice (SAMP8). In this study, they found that combined treatment exerted an additive restoring effect on learning and memory deficits in the MWM test and it synergistically reduced NFTs and expression of Amyloid precursor protein (APP) compared to either treatment alone in SAMP8 mice.

The possible combined effects of physical exercise and EE on experimental models have been explored in some studies. A study (51) showed that exposure to a EE including a voluntary running wheel could prevent spatial and visual memory impairment and neuronal apoptosis at a high altitude via VEGF signaling in hippocampal and visual cortex areas. In another study on experimentally induced cognitive deficits, it was indicated that a combined regimen of EE, nutrition, and physical exercise could markedly reverse spatial memory impairment and increase hippocampal neurogenesis in rats with lesioned brain (26). In studies on healthy animals, combination therapy with aerobic exercise and EE resulted in the best performance in memory tests and also the highest levels of molecular correlates of memory, including BDNF, NGF, and neurogenesis markers within the hippocampus (27, 28, 52, 53). Despite the wealth of research concerning the possible effect of single physical and cognitive training on AD behavioral and neural pathology in experimental models, to date, no study has addressed the potential of these two paradigms in combination.

Some papers have reviewed the benefits of combination therapy with physical and cognitive training for cognitive impairments in humans (24, 25, 54). A systematic review concluded that a combined paradigm of cognitive and physical training has superior benefits compared to individual stimuli for cognitive and functional status in older adults with and without cognitive deficiency (55). However, it was reported that while aerobic exercise alone could improve the verbal fluency of older adults, the addition of simultaneous cognitive activity did not provide synergistic enhancement in executive functions (56). These data support the assumption that simultaneous physical and cognitive training has advantages over either stimulus alone for enhancing behavioral and cognitive deficits associated with neurodegeneration and CNS insults. 

Our data revealed that the hippocampal DCX levels were significantly decreased in AD model rats, and although neither pretreatment with exercise nor EE alone restored it, combined pretreatment could significantly return it to the control levels. DCX is a brain-specific microtubule-associated protein that is involved in microtubule stabilization and nuclear translocation in migrating neuroblasts and young neuron DCX is found in the mitotic and early postmitotic neural cells, and it has a role in the neuronal growing processes (57, 58). Dramatic changes in synaptic architecture including loss of dendritic spines have been observed in AD animal models and the brains of AD patients (59). The enhancing effect of physical exercise and EE on hippocampal neurogenesis, a well-established mechanism for cognitive processes, has been widely documented in rodents (60).

It is difficult to conclude the exact correlated mechanisms for the additive effect of physical exercise and EE on cognitive impairments and hippocampal correlates of AD animals in our observations. It can be assumed that when exercise and EE are applied concurrently, the common potential mechanisms, such as neurogenesis, synaptic plasticity, increased expression of growth factors, increased blood follow, suppressing apoptosis, and balancing the oxidant-antioxidant system within the brain through which enhance the cognitive performances separately are amplified in combination therapy. The data from cognitively healthy animals show that simultaneous exercise and EE induce more new neurons and benefit brain function greater than either paradigm alone (27, 28). In rats with experimentally induced cognitive dysfunctions, Kapgal *et al*. (26) also reported that a combined paradigm of exercise, EE, and nutrition could overcome memory deficits and enhance hippocampal neurogenesis in the lesioned brain of rats. 

Animals were assigned to the different treatments from week 4 to week 8 and then subjected to intrahippocampal injection of Aβ 1-42 in week 9 of age. Spatial learning and memory in Morris water maze (MWM), inhibitory avoidance memory in passive avoidance task (PAT), and hippocampal extraction of neurogenesis markers were performed in week 10. For further details see methods section

EX: exercise; EE: enriched environment

Each day represents the mean latency (A) and distance moved (B) through four consecutive trials to find the hidden platform. There were no significant differences in spatial learning ability among the groups. (two-way repeated measure ANOVA)

(C) Spatial memory in MWM test in experimental groups. Spatial memory was defined as the percentage of time and distance spent in the target quadrant as well as the percentage of the number of crosses on this quadrant. The time and distance in the target quadrant were significantly decreased in the Alz group compared to the sham, Alz+EX, Alz+EE, and Alz+EX+EE groups

**P<0.01 indicating a difference from sham, #*P<*0.05 indicating a difference from Alz+EX and Alz+EE, •*P<*0.05 and •••*P<*0.001 represent the difference from Alz+EX+EE group. (D) Swimming speed in MWM. Data are shown as mean±SEM (one-way ANOVA followed by Tukey test)

The number of entries to the dark part was increased in the Alz group compared to the Alz+EE and Alz+EX+EE groups. **P<0.01 and ••*P<*0.01 indicating a difference from Alz+EX+EE and Alz+EE, respectively (A). Step-through latency (STL) to the dark part was decreased in the Alz group compared to the sham and Alz+EX+EE groups

**P<0.01 and ••*P<*0.01 indicating a difference from sham and Alz+EX+EE, respectively (B). The differences in Initial latency to the dark part (A) and in the total time in the dark compartment (TDC) (B) between the groups did not reach statistical significance

Data are shown as mean ± SEM (The Kruskal Wallis test followed by Bonferroni adjustment)

## Conclusion

In summary, intrahippocampal amyloid-beta infusion in the AD animal model caused deficits in recognition memory and hippocampal neurogenesis that resembled those described in AD patient pathology. Pretreatment with running exercise and EE could protect from memory impairments in AD rats, and these two factors in combination further led to a significantly more pronounced improvement in memory deficits and neurogenesis than either paradigm alone. Exercise alone may not be sufficient to prevent AD but in combination with other lifestyle habits such as cognitive engagement, it can provide a foundation for non-pharmacological interventions and a multidomain policy that may lead to a potential therapeutic strategy for humans suffering from AD. However, to apply these interventions to human subjects, more studies are needed to determine the benefits of physical exercise and EE alone or in combination and the probable mechanisms corresponding to these beneficial effects.

## Authors’ Contributions

V H and S S N designed the experiments; M S and M A K performed experiments and collected data; ES discussed the results and strategy; V H supervised, directed, and managed the study; V H, S S N, and, E S approved the final version of the manuscript. 

## Conflicts of Interest

The authors declare no conflicts of interest. 

## References

[1] Garre-Olmo J (2018). Epidemiology of alzheimer’s disease and other dementias. Rev Neurol.

[2] Nichols E, Szoeke CEI, Vollset SE, Abbasi N, Abd-Allah F, Abdela J (2019). Global, regional, and national burden of Alzheimer’s disease and other dementias, 1990-2016: A systematic analysis for the Global Burden of Disease Study 2016. Lancet Neurol.

[3] Nelson PT, Braak H, Markesbery WR (2009). Neuropathology and cognitive impairment in alzheimer disease: A complex but coherent relationship. J Neuropathol Exp Neurol.

[4] Williams BD, Pendleton N, Chandola T (2020). Cognitively stimulating activities and risk of probable dementia or cognitive impairment in the English Longitudinal Study of Ageing. SSM Popul Health.

[5] Alanko V, Udeh-Momoh C, Kivipelto M, Sandebring-Matton A (2022). Mechanisms underlying non-pharmacological dementia prevention strategies: A translational perspective. J Prev Alzheimer’s Dis.

[6] Herting MM, Chu X (2017). Exercise, cognition, and the adolescent brain. Birth Defects Res.

[7] Taskin S, Celik T, Demiryurek S, Turedi S, Taskin A (2022). Effects of different-intensity exercise and creatine supplementation on mitochondrial biogenesis and redox status in mice. Iran J Basic Med Sci.

[8] van Praag H (2009). Exercise and the brain: Something to chew on. Trends Neurosci.

[9] Liang KY, Mintun MA, Fagan AM, Goate AM, Bugg JM, Holtzman DM (2010). Exercise and Alzheimer’s disease biomarkers in cognitively normal older adults. Ann Neurol.

[10] Biazus-Sehn LF, Schuch FB, Firth J, Stigger F de S (2020). Effects of physical exercise on cognitive function of older adults with mild cognitive impairment: A systematic review and meta-analysis. Arch Gerontol Geriatr.

[11] Serra FT, Cardoso F dos S, Petraconi N, dos Santos JCC, Araujo BHS, Arida RM (2022). Resistance exercise improves learning and memory and modulates hippocampal metabolomic profile in aged rats. Neurosci Lett.

[12] Ryan SM, Kelly ÁM (2016). Exercise as a pro-cognitive, pro-neurogenic and anti-inflammatory intervention in transgenic mouse models of Alzheimer’s disease. Ageing Res Rev.

[13] Fancourt D, Steptoe A, Cadar D (2020). Community engagement and dementia risk: Time-to-event analyses from a national cohort study. J Epidemiol Community Health.

[14] Nousia A, Siokas V, Aretouli E, Messinis L, Aloizou AM, Martzoukou M (2018). Beneficial effect of multidomain cognitive training on the neuropsychological performance of patients with early-stage alzheimer’s disease. Neural Plast.

[15] Baroncelli L, Braschi C, Spolidoro M, Begenisic T, Sale A, Maffei L (2010). Nurturing brain plasticity: Impact of environmental enrichment. Cell Death Differ.

[16] Hu Y, Xu P, Pigino G, Brady ST, Larson J, Lazarov O (2010). Complex environment experience rescues impaired neurogenesis, enhances synaptic plasticity, and attenuates neuropathology in familial Alzheimer’s disease‐linked APPswe/PS1ΔE9 mice. FASEB J.

[17] Beauquis J, Pavía P, Pomilio C, Vinuesa A, Podlutskaya N, Galvan V (2013). Environmental enrichment prevents astroglial pathological changes in the hippocampus of APP transgenic mice, model of Alzheimer’s disease. Exp Neurol.

[18] Herring A, Ambrée O, Tomm M, Habermann H, Sachser N, Paulus W (2009). Environmental enrichment enhances cellular plasticity in transgenic mice with Alzheimer-like pathology. Exp Neurol.

[19] Liew AKY, Teo, CH Soga T (2022). The molecular effects of environmental enrichment on alzheimer’s disease. Mol Neurobiol.

[20] Petrosini L, De Bartolo P, Foti F, Gelfo F, Cutuli D, Leggio MG (2009). On whether the environmental enrichment may provide cognitive and brain reserves. Brain Res Rev.

[21] Arendash GW, Garcia MF, Costa DA, Cracchiolo JR, Wefes IM, Potter H (2004). Environmental enrichment improves cognition in aged Alzheimer’s transgenic mice despite stable β-amyloid deposition. Neuroreport.

[22] Jankowsky JL, Xu G, Fromholt D, Gonzales V, Borchelt DR (2003). Environmental enrichment exacerbates amyloid plaque formation in a transgenic mouse model of Alzheimer disease. J Neuropathol Exp Neurol.

[23] Mirochnic S, Wolf S, Staufenbiel M, Kempermann G (2009). Age effects on the regulation of adult hippocampal neurogenesis by physical activity and environmental enrichment in the APP23 mouse model of Alzheimer disease. Hippocampus.

[24] Karssemeijer Ega, Aaronson JA, Bossers WJ, Smits T, Olde Rikkert MGM, Kessels RPC (2017). Positive effects of combined cognitive and physical exercise training on cognitive function in older adults with mild cognitive impairment or dementia: A meta-analysis. Ageing Res Rev.

[25] Meng Q, Yin H, Wang S, Shang B, Meng X, Yan M (2022). The effect of combined cognitive intervention and physical exercise on cognitive function in older adults with mild cognitive impairment: a meta-analysis of randomized controlled trials. Aging Clin Exp Res.

[26] Kapgal V, Prem N, Hegde P, Laxmi TR, Kutty BM (2016). Long term exposure to combination paradigm of environmental enrichment, physical exercise and diet reverses the spatial memory deficits and restores hippocampal neurogenesis in ventral subicular lesioned rats. Neurobiol Learn Mem.

[27] Fabel K, Wolf SA, Ehninger D, Babu H, Leal-Galicia P, Kempermann G (2009). Additive effects of physical exercise and environmental enrichment on adult hippocampal neurogenesis in mice. Front Neurosci.

[28] Khodadadegan MA, Negah SS, Saheb M, Gholami J, Arabi MH, Hajali V (2021). Combination effect of exercise and environmental enrichment on cognitive functions and hippocampal neurogenesis markers of rat. Neuroreport.

[29] Saadati H, Sheibani V, Esmaeili-Mahani S, Darvishzadeh-Mahani F, Mazhari S (2014). Prior regular exercise reverses the decreased effects of sleep deprivation on brain-derived neurotrophic factor levels in the hippocampus of ovariectomized female rats. Regul Pept.

[30] Kim DY, Jung SY, Kim K, Kim CJ (2016). Treadmill exercise ameliorates Alzheimer disease-associated memory loss through the Wnt signaling pathway in the streptozotocin-induced diabetic rats. J Exerc Rehabil.

[31] Abubakar MB, Sanusi KO, Ugusman A, Mohamed W, Kamal H, Ibrahim NH (2022). Alzheimer’s disease: An update and insights into pathophysiology. Front Aging Neurosci.

[32] LaFerla FM, Green KN (2012). Animal models of Alzheimer disease. Cold Spring Harb Perspect Med.

[33] Facchinetti R, Bronzuoli MR, Scuderi C (2018). An animal model of alzheimer disease based on the intrahippocampal injection of amyloid β-peptide (1–42). Methods Mol Biol.

[34] Prado Lima MG, Schimidt HL, Garcia A, Daré LR, Carpes FP, Izquierdo I (2018). Environmental enrichment and exercise are better than social enrichment to reduce memory deficits in amyloid beta neurotoxicity. Proc Natl Acad Sci U S A.

[35] Dare LR, Garcia A, Soares CB, Lopes L, Neves BHS, Dias DV (2020). The reversal of memory deficits in an alzheimer’s disease model using physical and cognitive exercise. Front Behav Neurosci.

[36] Pardon MC, Sarmad S, Rattray I, Bates TE, Scullion GA, Marsden CA (2009). Repeated novel cage exposure-induced improvement of early Alzheimer’s-like cognitive and amyloid changes in TASTPM mice is unrelated to changes in brain endocannabinoids levels. Neurobiol Aging.

[37] Görtz N, Lewejohann L, Tomm M, Ambrée O, Keyvani K, Paulus W (2008). Effects of environmental enrichment on exploration, anxiety, and memory in female TgCRND8 Alzheimer mice. Behav Brain Res.

[38] Herring A, Lewejohann L, Panzer AL, Donath A, Kröll O, Sachser N (2011). Preventive and therapeutic types of environmental enrichment counteract beta amyloid pathology by different molecular mechanisms. Neurobiol Dis.

[39] McGurran H, Glenn JM, Madero EN, Bott NT (2019). Prevention and treatment of Alzheimer’s disease: Biological mechanisms of exercise. J Alzheimers Dis.

[40] Farì G, Lunetti P, Pignatelli G, Raele MV, Cera A, Mintrone G (2021). The effect of physical exercise on cognitive impairment in neurodegenerative disease: From pathophysiology to clinical and rehabilitative aspects. Int J Mol Sci.

[41] Sattler C, Toro P, Schönknecht P, Schröder J (2012). Cognitive activity, education and socioeconomic status as preventive factors for mild cognitive impairment and Alzheimer’s disease. Psychiatry Res.

[42] Arenaza-Urquijo EM, Wirth M, Chételat G (2015). Cognitive reserve and lifestyle: Moving towards preclinical Alzheimer’s disease. Front Aging Neurosci.

[43] Cardoso F dos S, França EF, Serra FT, Victorino AB, de Almeida AA, Fernandes J (2017). Aerobic exercise reduces hippocampal ERK and p38 activation and improves memory of middle-aged rats. Hippocampus.

[44] Hullinger R, O’Riordan K, Burger C (2015). Environmental enrichment improves learning and memory and long-term potentiation in young adult rats through a mechanism requiring mGluR5 signaling and sustained activation of p70s6k. Neurobiol Learn Mem.

[45] Kim BK, Shin MS, Kim CJ, Baek SB, Ko YC, Kim YP (2014). Treadmill exercise improves short-term memory by enhancing neurogenesis in amyloid beta-induced Alzheimer disease rats. J Exerc Rehabil.

[46] Dao AT, Zagaar MA, Levine AT, Salim S, Eriksen JL, Alkadhi KA (2013). Treadmill exercise prevents learning and memory impairment in Alzheimer’s disease-like pathology. Curr Alzheimer Res.

[47] Jahangiri Z, Gholamnezhad Z, Hosseini M (2019). Neuroprotective effects of exercise in rodent models of memory deficit and Alzheimer’s. Metab Brain Dis.

[48] Cho JY, Um HS, Kang EB, Cho IH, Kim CH, Cho JS (2010). The combination of exercise training and α-lipoic acid treatment has therapeutic effects on the pathogenic phenotypes of Alzheimer’s disease in NSE/APPsw-transgenic mice. Int J Mol Med.

[49] Li W yi, Gao J yan, Lin SY, Pan S tao, Xiao B, Ma Y tao (2022). Effects of involuntary and voluntary exercise in combination with acousto-optic stimulation on adult neurogenesis in an Alzheimer’s mouse model. Mol Neurobiol.

[50] Dong J, Zhou M, Wu X, Du M, Wang X (2012). Memantine combined with environmental enrichment improves spatial memory and alleviates Alzheimer’s disease-like pathology in senescence-accelerated prone-8 (SAMP8) mice. J Biomed Res.

[51] Koester-Hegmann C, Bengoetxea H, Kosenkov D, Thiersch M, Haider T, Gassmann M (2019). High-altitude cognitive impairment is prevented by enriched environment including exercise via VEGF signaling. Front Cell Neurosci.

[52] Santoso DII, Yolanda S, Redjeki S, Andraini T, Ivanali K (2020). Continuous environmental enrichment and aerobic exercise improves spatial memory: Focus on rat hippocampal BDNF and NGF. Comp Exerc Physiol.

[53] Aguspa Dita DA, Paramita N, Kodariah R, Kartinah NT (2013). Environmental enrichment and aerobic exercise enhances spatial memory and synaptophysin expression in Rats. Indones Biomed J.

[54] Zhao X, Huang X, Cai Y, Cao T, Wan Q (2022). The relative effectiveness of different combination modes for exercise and cognitive training on cognitive function in people with mild cognitive impairment or Alzheimer’s disease: A network meta-analysis. Aging Ment Health.

[55] Law LLF, Barnett F, Yau MK, Gray MA (2014). Effects of combined cognitive and exercise interventions on cognition in older adults with and without cognitive impairment: A systematic review. Ageing Res Rev.

[56] Nocera JR, Mammino K, Kommula Y, Wharton W, Crosson B, McGregor KM (2020). Effects of combined aerobic exercise and cognitive training on verbal fluency in older adults. Gerontol Geriatr Med.

[57] Koizumi H, Higginbotham H, Poon T, Tanaka T, Brinkman BC, Gleeson JG (2006). Doublecortin maintains bipolar shape and nuclear translocation during migration in the adult forebrain. Nat Neurosci.

[58] Friocourt G, Koulakoff A, Chafey P, Boucher D, Fauchereau F, Chelly J (2003). Doublecortin functions at the extremities of growing neuronal processes. Cereb Cortex.

[59] Yu W, Lu B (2012). Synapses and dendritic spines as pathogenic targets in Alzheimer’s disease. Neural Plast.

[60] Baptista P, Andrade JP (2018). Adult hippocampal neurogenesis: Regulation and possible functional and clinical correlates. Front Neuroanat.

